# Patient satisfaction with peri-partum care at Bertha Gxowa district hospital, South Africa

**DOI:** 10.4102/phcfm.v12i1.2409

**Published:** 2020-08-13

**Authors:** Nonhlanhla Khumalo, Edrone Rwakaikara

**Affiliations:** 1 Department of Family Medicine and Primary Care, Faculty of Health Sciences, University of the Witwatersrand, Johannesburg, South Africa

**Keywords:** patient satisfaction, peri-partum, care, family medicine, primary care, general practice, maternal health

## Abstract

**Background:**

Patient satisfaction is one of the key outcome measures of healthcare services.

**Aim and Setting:**

To explore factors that influence women’s satisfaction with peri-partum care at Bertha Gxowa district hospital, South African primary care.

**Methods:**

A cross-sectional study involving 260 women was conducted. A structured questionnaire collected information from participants on pain relief, health education provided by healthcare providers, privacy, cleanliness of the ward and their participation in decision-making about care received in the peri-partum period.

**Results:**

Most respondents were co-habiting with their partners (100, 38%) and had completed only secondary school education (119, 46%). The average participant age was 27 years, with an average parity of two children. Most participants were satisfied with the privacy (218, 84%) and the general cleanliness of the wards (233, 90%). However, large proportions of women were dissatisfied with the information given to them by doctors (104, 55%) and nurses (89, 37%), and the rest were unsure. About 189 (73%) participants were dissatisfied with the extent of their participation in decision-making about their own care. The study had a caesarean rate of 53 (20%). Compared to normal vaginal delivery, participants who had caesarean section were significantly more likely to report being satisfied with pain relief during labour (*p* < 0.001).

**Conclusion:**

The study findings showed varying levels of satisfaction with different aspects of peri-partum care and suggested the need for better pain relief during vaginal delivery, information sharing by doctors and patient emancipation for decision-making about their own care.

## Introduction

Patient satisfaction is a concept that has been in vogue for many years and has been a subject of research in different countries. Although each researcher has defined it in his or her own way, the consensus amongst most researchers is that patient satisfaction involves the correlation between what the patient expects and what the healthcare environment actually delivers.^1,2,3,4^ Previous studies on patient satisfaction show that factors that influence client satisfaction with regard to care involve satisfaction with the overall health service, health workers’ communication, health workers’ attitude and the hospital environment.^5,6^ Many countries, including South Africa, have therefore designed strategies that focus on patients’ needs and better communication to improve patient satisfaction. One of the strategies implemented in South Africa to achieve this was the introduction of the Bath-Pele principles. The aim of this strategy was to improve the quality and accessibility of government services and to increase accountability of the healthcare providers.^7,8^ While these principles were generic across public service platforms, they also govern the delivery of healthcare services, including maternity care.

Studies on patient satisfaction and the influence of socio-demographic factors have reported varying findings. While some have found that patients from low socio-economic status with low level of education tend to have higher level of satisfaction,^6,9,10^ others have found that immigrants from low- and middle-income countries and citizens with high level of education report high rates of satisfaction.^9^ The authors suggested that immigrants from low-income countries were likely to have low expectations and were likely to appreciate whatever service was offered to them.^6,9^ However, other studies have also found that socio-demographic factors had little or no impact on women’s satisfaction with maternity care.^2,11,12^

Areas of dissatisfaction with labour in some African countries were poor pain control and drug prescription during labour in Ethopia^5^ and Limpopo,^13^ while Eritrea and South African districts and provinces recorded dissatisfaction mostly because of abuse and disrespect, health system failures and accountability issues, as well as lack of communication between providers and clients. However, in an extensive systematic review of 137 studies, pain and pain relief did not play major roles in satisfaction with the childbirth experience, unless expectations regarding pain and pain relief were not met.^2^

Communication between health workers and patients is important and is assured by information giving and sharing, and it has been shown to influence patient satisfaction.^3^ In Ghana, a cross-sectional study of 885 women with normal vaginal deliveries looked at the association between experiences during childbirth and satisfaction with childbirth service. It found that amongst other predictors of dissatisfaction, women who felt that they were not given enough information about their condition and care were 9.4 times more likely to be dissatisfied with childbirth care than women who felt that they were given the right amount of information.^14^

Peri-partum care services are priority health programmes in South Africa, and they reflect the quality of care rendered in the public healthcare system. The principal objective of maternity care was to make the entire labour process a positive experience for the women and their families, and to ensure that the birth process proceeds with minimum or no complications.^8^ Central to these is that women should be satisfied with the services received during labour. In South Africa, pregnant women are expected to deliver in primary health care (PHC) facilities, which are headed by midwives, for which the district hospital is the referral centre.^15^ Patient satisfaction is a composite measure of the health system’s performance^5,6^ and whether the findings of studies conducted in other contexts are applicable to maternity services in the Ekurhuleni district PHC setting is unknown. The aim of this study was therefore to assess patient satisfaction and determine factors that influence it during the peri-partum period at a district hospital in Ekurhuleni health district, southeast of Johannesburg, South Africa.

## Methods

### Design and study sites

This was a quantitative, cross-sectional study of women attending the third day postpartum clinic at Bertha Gxowa district hospital. This hospital situated in Germiston, south-east of the city of Johannesburg, South Africa, is the only district hospital in the Ekurhuleni health district. The catchment population is women who deliver their babies in the hospital over 2 months during the study period.^16^ There is no Midwife Obstetric Unit (MOU) in the Germiston area, and hence, normal deliveries are done in hospital. This is reflected in the average number of 450–500 deliveries per month, which is pretty high for a district hospital.^16^ The maternity unit in a district hospital such as Bertha Gxowa comprises a team that includes midwives, doctors, professional nurses and the general staff.

Women who deliver in the hospital are seen with their babies for the third day review in the hospital. Those that delivered by normal vaginal delivery are seen in the antenatal clinic (ANC), which is divided into prenatal and postnatal areas. Those who delivered by caesarean section are seen in the postnatal ward.

The study was conducted at two sites within the hospital:

- The ANC-postnatal side: for women who had a normal vaginal delivery and were returning for their third day follow-up visits. This visit caters for women who have delivered up to 10 days.
- The postnatal ward: for women who had elective or emergency caesarean. Women who had caesarean operation with no complications are discharged on the third day post-operation for follow-up at their local clinics.

### Samples and procedure

The sample size was calculated to be 260 using the Raosoft software.^17^ This calculation assumed a 5% margin of error, 95% confidence interval, a response distribution rate of 50% and an estimated 800 women delivering over 2 months of study. The eligible population was all women aged over 18 years who delivered their babies in Bertha Gxowa district hospital and presented for the 3 days postnatal visit or was third day post caesarean section in the postnatal.

#### Procedure

Non-randomised, convenient sampling was used for both study sites. Thirty-two women were sampled every week for 7 weeks, and 36 were sampled on the eighth week to reach a total of 260. The eligible population was women in the ward on their third day after caesarean section were all invited to participate in the study because the number of caesarean operations was small. The number of caesarean operations varied between zero and three per day. Every woman in the postnatal review clinic was eligible to participate in the study after they were seen by the nurses. As soon as the interview with one woman was completed, the next available patient was invited to participate in the study until the number for the day was reached.

Recruitment was done on every day of the week till the sample size was attained over a period of 2 months. The ratio of caesarean sections to vaginal deliveries was 53 (20%) to 207 (80%).

#### Data collection tool

Data collection was performed using a structured questionnaire, which was derived from two previously used questionnaires that were adapted to our South African setting. Some questions came from the Sylheti Questionnaire and some questions came from the COMFORT scale on maternal satisfaction with peri-partum services. A structured questionnaire combining the two studies was developed *de novo* based on the literature.^18,19^ Pilot study was conducted in five women and no corrections were made to the questionnaire.

Patients who accepted to participate were interviewed by the lead researcher, and their responses were recorded on the data collection tool. The questionnaire used a Likert Scale to rate responses from participants and had five sections: The first section collected information on demographic characteristics, and the second section collected information on the mode of delivery. In the third and fourth sections, participants were asked to rate their level of satisfaction with different aspects of care received on a scale of 5, ranging from 1 = strongly agree to 5 = strongly disagree, with regard to pain relief, cleanliness, privacy, delivery care, information given to them during labour and participation in their care.

### Data analysis

Data were captured onto Microsoft Excel, cleaned and then imported to STATA-10 statistical software for analysis. Descriptive analysis was performed, and participants were described using frequencies and proportions for categorical data, and means with standard deviations for numeric data. Chi-square test and *t*-test were used to test for associations between mode of delivery and satisfaction with pain relief during childbirth. Statistical significance was set at *p* < 0.05. Main outcome measures included the proportions of participants satisfied with the privacy, cleanliness of the ward, the information given to them by healthcare providers, the extent of their participation in decision-making about their own care, and socio-demographic and clinical factors significantly associated with being satisfied with aspects of care.

### Ethical consideration

Approval to conduct the study was obtained from the hospital chief executive officer, the Ekurhuleni District Ethical Committee and the Human Research Ethics Committee of the University of the Witwatersrand. The ethical clearance number is M111014. Written and signed informed consent was obtained from the respondents who took part in the study.

## Results

Most of the participants (103, 40%) were aged between 18 and 24 years; 227 (87%) participants completed secondary school education. The mean age was 27 years with average parity of two children. Only 62 (24%) of the participants were married, while 33 (13%) participants had tertiary education. There were 53 (20%) women participants who delivered by caesarean section.

Table 1 shows satisfaction with pain relief and indicates that 83 (32%) and 94 (36%) of the participants were satisfied with what was done to relieve pain during and after, respectively.

**TABLE 1:** Satisfaction with pain relief during labour.

Variable	Total (*n* = 260)	
	*n*	%
**During labour, the nurses respected your description of pain**		
Disagree/strongly disagree	192	73
Not sure	7	3
Agree/strongly agree	61	24
**You were satisfied with what was done to relieve pain during labour?**		
Disagree/strongly disagree	176	68
Not sure	1	0
Agree/strongly agree	83	32
**You were satisfied with what was done to relieve pain after you delivered your baby?**		
Disagree/strongly disagree	164	63
Not sure	2	1
Agree/strongly agree	94	36

Only 64 (24%) participants reported that nurses respected their personal description (assessment) of pain.

The proportion of participants who responded to the question on environmental factors, which included privacy and cleanliness, is shown in Table 2. Most participants reported that clinical and non-clinical staff respected their privacy during labour. Only 79 (30%) participants felt that there were ‘a lot of people around them during labour’. Most of the participants reported that their beddings (252, 97%), rooms (244, 93%) and toilets (233, 90%) were clean. In Table 3, 166 (64%) participants expressed satisfaction with hospital care and indicated they were satisfied with the time spent on learning about self-care in the postpartum period. There were varying levels of dissatisfaction, with only 110 (43%) participants being satisfied with time spent on explaining what was done and why?, 102 (39%) participants being satisfied with listening to their needs and 104 (40%) participants being satisfied with time spent waiting for nurses to respond to a need.

**TABLE 2:** Satisfaction with environmental factors: Privacy and cleanliness.

Variable	Total (*n* = 260)	
	*n*	%
**When you delivered your baby, the curtains were closed**		
(53 women delivered by caesarean section)		
Disagree/strongly disagree	75	36
Not sure	7	3
Agree/strongly agree	125	61
**During labour, you felt like there were a lot of people around**		
Disagree/strongly disagree	136	66
Not sure	9	3
Agree/strongly agree	79	31
**The medical staff respected your privacy, you were not left exposed**		
Disagree/strongly disagree	96	37
Not sure	2	1
Agree/strongly agree	162	62
**Non-medical staff (porters, cleaners, etc.) respected your privacy**		
Disagree/strongly disagree	41	16
Not sure	1	0
Agree/strongly agree	218	84
**The toilets were clean**		
Disagree/strongly disagree	24	9
Not sure	3	1
Agree/strongly agree	233	90
**The delivery room was clean**		
Disagree/strongly disagree	9	4
Not sure	7	3
Agree/strongly agree	244	94
**The bedding was clean**		
Disagree/strongly disagree	7	3
Not sure	1	0
Agree/strongly agree	252	97

**TABLE 3:** Satisfaction with hospital care.

Variable	Total (*n* = 260)	
	*n*	%
**Satisfied with the time that was spent explaining about what was done and why.**		
Disagree/strongly disagree	149	57
Not sure	1	0
Agree/strongly agree	110	43
**Satisfied with the time spent listening to your needs.**		
Disagree/strongly disagree	158	61
Not sure	0	0
Agree/strongly agree	102	39
**Satisfied with the time you spent waiting for nurses to respond to your needs.**		
Disagree/strongly disagree	153	59
Not sure	3	1
Agree/strongly agree	104	40
**Satisfied with the time spent teaching you in the postpartum period to care for yourself.**		
Disagree/strongly disagree	90	34
Not sure	4	2
Agree/strongly agree	166	64

In Table 4, although most of the participants (208, 80%) were satisfied with the consistency of information received from healthcare providers, 162 (63%) versus 80 (43%) participants felt doctors were less likely than nurses to give information required for informed choices on self-care.

**TABLE 4:** Satisfaction with information sharing by doctors and nurse.

Variable	Total (*n* = 260)	
	*n*	%
**Doctors gave you information you needed to make informed choices about your care**		
Disagree/strongly disagree	104	55
Not sure	3	2
Agree/strongly agree	80	43
**Nurses gave you information you needed to make informed choices about your care**		
Disagree/strongly disagree	89	34
Not sure	9	3
Agree/strongly agree	162	63
**The information you received from different health providers was consistent**		
Disagree/strongly disagree	50	19
Not sure	2	1
Agree/strongly agree	208	80

Few participants (67, 26%) were satisfied with their level of involvement in their own care, and 75 (29%) participants were satisfied with the degree to which healthcare workers supported their decisions. A total of 137 (53%) participants felt free to ask questions, 175 (67%) participants did not feel pressurised to agree with health providers’ management plan and 154 (59%) participants, if they were to have another baby, would return to the hospital, as shown in Table 5.

**TABLE 5:** Satisfaction with involvement in their own care.

Variable	Total (*n* = 260)	
	*n*	%
**Satisfied with the number of times doctors/nurses asked for your opinion in planning your care.**		
Disagree/strongly disagree	189	73
Not sure	4	1
Agree/strongly agree	67	26
**Satisfied with the degree to which caregivers supported your decision**		
Disagree/strongly disagree	179	69
Not sure	6	2
Agree/strongly agree	75	29
**You felt free to ask questions.**		
Disagree/strongly disagree	123	47
Not sure	0	0
Agree/strongly agree	137	53
**You felt pressured to agree with care givers management plan/treatment.**		
Disagree/strongly disagree	175	67
Not sure	5	2
Agree/strongly agree	80	31
**If you were to have another baby, you would return to this hospital.**		
Disagree/strongly disagree	97	38
Not sure	9	3
Agree/strongly agree	154	59

Table 6 shows the association between the mode of delivery (normal vaginal delivery or caesarean section) with satisfaction and pain relief during childbirth. There was an association between the mode of delivery with satisfaction and pain relief during childbirth. Of concern is the finding that most participants (76%) were dissatisfied with pain relief during labour, and this was more prevalent in women who delivered by normal vaginal delivery than in women who had caesarean section (76% vs. 32%). Women who delivered by caesarean section were significantly more likely to report being satisfied with pain relief during labour (*p* < 0.001).

**TABLE 6:** Association between mode of delivery and satisfaction with pain relief.

Variable	*n*	%	Association (%)
**Normal vaginal delivery and satisfaction with pain relief during labour**			
Disagree/strongly disagree	159	61	76
Agree/strongly agree	48	19	24
**Caesarean section and satisfaction with pain relief**			
Disagree/strongly disagree	17	6	32
Agree/strongly agree	36	14	68
Pearson’s chi-square test (2) = 39.5890, Pr = 0.000			

## Discussion

This study assessed patient satisfaction with peri-partum care and found varying levels of satisfaction with different aspects of care and indicated high levels of satisfaction with care during child birth, irrespective of age, marital status and the level of education. The participants reported higher levels of satisfaction with the environmental factors such as cleanliness of the environment (93%) and how privacy was maintained by both medical and non-medical staff (84%). These findings concur with the results of an extensive systematic review completed by Srivastava for 54 studies conducted in different developing countries where they looked at all dimensions of care during childbirth and found that cleanliness of the environment and privacy greatly influenced satisfaction.^4^ On the contrary, studies conducted in other African countries like Ethiopia and Eritrea, and other developing countries such as Sri Lanka, found that women were not satisfied with the level of cleanliness and privacy during labour.^3,5,20^ Some of these studies attributed their findings to the fact that these deliveries occurred in teaching hospitals with lots of students present, limiting the level of privacy.^6^ However, the findings of this study are more likely because cleanliness, respecting patients’ privacy and their dignity are one of the six key foci of the National Core Standards in improving the quality of the public health sector services in South Africa.^21^ As such, it is assessed daily by managers and supervisors, and randomly by independent external auditors.^21^ Hence, political commitment to the desired key outcome of healthcare may assist in the achievement of such outcomes at the operational level. Alternatively, an assumption can be made that intensive training of public service workers in the Batho-pele principles might have resulted in hospital personnel providing respectful care to their patients by maintaining privacy and cleanliness of the environment.^7^ Summed up, the findings of this study reiterate the importance of environmental factors as major contributing factors affecting satisfaction with childbirth.^3,5^

This may be attributed to the clinician’s awareness of the importance of pain relief after caesarean section, and its importance in assisting patients in management and early mobilisation, thereby facilitating the healing process, as stated in the findings of this study, which was conducted in South Africa and involved 31 general practitioners who were involved in the care of caesarean section patients.^22^ In their report, the authors suggested that the availability of facility guidelines and protocols as well as the availability of analgesic drugs were some of the factors that influence doctors’ prescriptions for caesarean postoperative pain management.^22^ The influence of the availability of analgesic drugs on prescription not only influences doctors but also affects midwives’ behaviour, as shown in another study conducted in Tshwane, South Africa, which found that women were not given pain relievers by midwives during normal vaginal delivery, in spite of guidelines and protocols, because of the unavailability of pain relief medication in four of the five Midwives Obstetric Units studied.^23^ However, this is not acceptable as there are provisions and procedures to follow in the event of medical stock-outs. There is therefore a need to ensure that the necessary analgesic drugs are available, and managers who do not order drugs should be held accountable.

Another possible explanation for the lack of pain relief during labour is the healthcare personnel’s attitude based on cultural and religious beliefs that pain is a normal process of labour, and it is something to be endured by all women in labour, as it enhances the bond between the mother and the child.^24,25^ Such beliefs have been reported in a Ghanaian study, where midwives believed that pain during labour was brought on by God and should not be tampered with.^26^ The findings of this study showed that midwives who were Christians believed that labour pains were a curse from God, and women needed to pray to God to ease their pain.^26^ The idea of not providing pain relief in childbirth is also influenced by other beliefs as found in a bibliographic review of cultural influences on attitudes of women and health professionals regarding pain in labour.^27^ There were varying beliefs for and against provision of analgesics during childbirth. Studies that were against pain relief spoke of the oppressive character of medicalisation of childbirth because it emphasises drugs as the only means of controlling pain and denied women their right to express feelings of pain that only a woman in labour can experience.^27^ According to the South African maternity guidelines, pethidine and promethazine are to be offered to all women, and only those that sought permission are prescribed the drugs in labour intramuscularly, this was not done in this study.^17^ The midwives could have been influenced by their fear of administering pethidine in labour because of anxiety about side effects of the drug as was noted in Ghana where they did not administer the appropriate dose of pethidine in spite of the doctor’s prescription.^27,28^ In this study, midwives relied more on non-pharmacological ways of relieving labour pains.^28^ The midwives in this study might also share the same cultural or religious belief system, hence their reluctance to provide pain relief. This indicates that the maternity unit was not following the national guidelines, which clearly states that analgesics should be used to relieve pain during labour and after delivery. Such beliefs are contrary to expectations that midwives should play a positive role with regard to women in labour by caring and encouraging the women experiencing labour pain, and managers should ensure proper utilisation of available guidelines.^26^

Lack of any pain relief during labour is unacceptable and is remembered long after childbirth, as shown in longitudinal studies conducted in Sweden and Japan, which showed that the memory of labour pain did not diminish over time in women with negative overall experience of childbirth.^29,30^ There are also other barriers that have been reported as impediments to the provision of analgesics during childbirth – these include understaffing, overwork and delivering many women at the same time, all of which prevent midwives from administering analgesics to women in labour.^14^ There is an acute shortage of health personnel in most public hospitals, and Bertha Gxowa district hospital is no exception, as it is not uncommon for one midwife to care for more women simultaneously in labour, as in most developing countries.^3,31^ While these cannot be justified, they have clinical implications and warrant a population norm-driven recruitment of clinical staff to bridge staff shortages.^14^ There is a greater need to humanise the birth process and to change the culture of denying women analgesics during the labour process, and this can be done only through continuous monitoring and training of staff. It should include women’s choices on analgesia during childbirth and the labour process, and can be done through continuous monitoring and training of staff while using patients’ feedback.^32^

South Africa is a diverse country with 11 official languages.^33^ The study found that patients were less satisfied with the information given to them by the doctors, compared to midwives. These results are consistent with the finding of the study by Kathy et al. on midwifery provider communication in maternity decisions. They reported that patients found it easier to communicate with midwives who used less medical jargon when communicating with patients than doctors.^34^ This could be the case in this study. Alternatively, the dissatisfaction might be attributable to the fact that most doctors working in the maternity unit at the time of the study were mostly male international medical graduates (IMGs) and therefore not fluent in the local languages. On the other hand, most nurses were female South Africans and therefore fluent in the local languages spoken in the area. The disparities between these groups has the potential for miscommunication and poor understanding between the IMGs and their patients, reiterating the need for a comprehensive programme that promotes IMGs proficiency in local languages, including sensitivity to gender and cultural differences. Women are more likely to relate to other women in labour and therefore more likely to empathise.^28^ However, this may not be necessarily true in all settings, as shown by Mthombeni et al. in a study conducted in the Limpopo Province of South Africa, where male nurses were found to be more caring and sympathetic to women in pain and communicated well and maintained privacy when examining women in labour.^35^ The authors attributed this to women’s perception that men do not have preconceived ideas about their childbirth experience. According to the *South African National Health Act*, the healthcare providers should inform healthcare users of their health status in a language the user understands.^36^ Poor communication with patients or lack thereof creates ethical dilemma where patient’s autonomy may not be respected. Decisions about the patient’s care are then made without the patient’s clear consent and involvement because the patient has no or poor understanding of the intended care process, increasing patient dissatisfaction.^3^ This is emphasised by the findings of a Spanish study, which was conducted in two university hospitals, that found communication and information sharing as important aspects of professional support during labour and that patients came with this expectation.^37,38,39^ It is therefore important for the IMG to learn at least one commonly used local language so as to improve the odds of patient satisfaction with their care.

This study indicates that participants were not satisfied with hospital care and their level of participation in their own care – in particular, the time spent explaining what was done (and why), listening to patients’ needs and the waiting for clinicians to respond. These findings are consistent with a study conducted in the Eastern Cape, South Africa, by the Human Rights Commission in which women were not satisfied with the care that they received during labour because the nurses did not respond to their needs.^14^ It appeared there was no time on the part of the caregivers to explain, to listen to or to attend to women in labour. In spite of participants’ overall high levels of dissatisfaction, most respondents (60%) reported that they would return to the hospital if they were to have another baby. This may be because of the lack of choice in available birthing facilities because Bertha Gxowa is the only public hospital in the study area. Alternatively, the survival of the labour process and having a healthy baby may be seen as enough reasons to warrant returning back to have future babies and/or recommending the facility to others.

This study has some limitations: the convenience sampling method could have introduced sampling bias, limiting the generalisation of the study findings. Also, the use of self-reports may result in information bias because the respondents may be reluctant to criticise the care received because of fear of reprimand and the perception that they needed to show gratitude. However, this was addressed by encouraging participants to be as honest as possible and assuring that their responses would not influence the quality of service received but will only be used for the research and quality improvement purposes. Notwithstanding these potential limitations, this study provides one of the few insights into patients’ experience of maternity care in South African primary care setting and has the potential to inform interventions aimed at improving maternity care in this and similar contexts.

## Conclusion

There were varying levels of satisfaction with aspects of care received by participants during the peri-partum period. Satisfaction with cleanliness, privacy and information sharing by nurses were reportedly high. In contrast, satisfaction with pain relief, time spent explaining procedures and information sharing by doctors was a matter of concern. These factors should be considered when designing quality improvement programmes to ensure respectful and patient-centred maternity care in MOUs in South Africa and in similar settings elsewhere. Considering the limitations, better designed quantitative and even qualitative studies are warranted to deepen our understanding of patient experience and satisfaction with peri-partum care.
